# Structural Basis for Par-4 Recognition by the SPRY Domain- and SOCS Box-Containing Proteins SPSB1, SPSB2, and SPSB4

**DOI:** 10.1016/j.jmb.2010.06.017

**Published:** 2010-08-20

**Authors:** Panagis Filippakopoulos, Andrew Low, Timothy D. Sharpe, Jonas Uppenberg, Shenggen Yao, Zhihe Kuang, Pavel Savitsky, Rowena S. Lewis, Sandra E. Nicholson, Raymond S. Norton, Alex N. Bullock

**Affiliations:** 1 Structural Genomics Consortium, University of Oxford, Old Road Campus, Roosevelt Drive, Oxford OX3 7DQ, UK; 2 Walter and Eliza Hall Institute of Medical Research, 1G Royal Parade, Parkville, Victoria 3052, Australia

**Keywords:** HSQC, heteronuclear single quantum coherence, SPSB, SPRY domain- and SOCS box-containing protein, hPar-4, human prostate apoptosis response protein-4, hSPSB, human SPSB, ITC, isothermal titration calorimetry, mSPSB, murine SPSB, PDB, Protein Data Bank, GST, glutathione *S*-transferase, PEG, polyethylene glycol, X-ray crystallography, NMR, ITC, protein structure, protein–peptide interaction

## Abstract

The mammalian SPRY domain- and SOCS box-containing proteins, SPSB1 to SPSB4, belong to the SOCS box family of E3 ubiquitin ligases. Substrate recognition sites for the SPRY domain are identified only for human Par-4 (ELNNNL) and for the *Drosophila* orthologue GUSTAVUS binding to the DEAD-box RNA helicase VASA (DINNNN). To further investigate this consensus motif, we determined the crystal structures of SPSB1, SPSB2, and SPSB4, as well as their binding modes and affinities for both Par-4 and VASA. Mutation of each of the three Asn residues in Par-4 abrogated binding to all three SPSB proteins, while changing EL to DI enhanced binding. By comparison to SPSB1 and SPSB4, the more divergent protein SPSB2 showed only weak binding to Par-4 and was hypersensitive to DI substitution. Par-4_(59–77)_ binding perturbed NMR resonances from a number of SPSB2 residues flanking the ELNNN binding site, including loop D, which binds the EL/DI sequence. Although interactions with the consensus peptide motif were conserved in all structures, flanking sites in SPSB2 were identified as sites of structural change. These structural changes limit high-affinity interactions for SPSB2 to aspartate-containing sequences, whereas SPSB1 and SPSB4 bind strongly to both Par-4 and VASA peptides.

## Introduction

The SPRY domain- and SOCS box-containing proteins, SPSB1 to SPSB4 (also known as SSB-1 to -4), contain a protein interaction domain known as the SPRY domain, which was first identified as a sequence repeat in the dual-specificity kinase splA and ryanodine receptors,^1^ together with a C-terminal SOCS box motif.^2^ The presence of the SOCS box motif suggests that the SPSB proteins may function as part of an E3 ubiquitin ligase, with the SPRY domain determining the substrate(s) for ubiquitination,^3^ and this has been confirmed recently for SPSB2 (Z.K., R.S.L., R.S.N., and S.E.N., personal communication). All four SPSB proteins also interact with c-Met, the hepatocyte growth factor receptor,^4^ and SPSB1, SPSB2, and SPSB4, but not SPSB3, interact with human prostate apoptosis response protein-4 (hPar-4).^5^ hPar-4 is up-regulated in prostate cancer cells undergoing apoptosis^6^ and appears to be a regulator of the ζPKC–NF-κB pathway, with Par-4 null mice showing enhanced T-cell proliferation and tumor formation, primarily through increased NF-κB signalling and resistance to apoptosis.^7,8^ The *Drosophila* SPSB protein homologue, GUSTAVUS, interacts with the DEAD-box RNA helicase VASA.^9^ The identification of similar sequences in hPar-4 (ELNNNL) and VASA (DINNNN)^10^ suggests that the SPSB1, SPSB2, and SPSB4 SPRY domains may recognize a common peptide epitope in these proteins.

There are currently > 1600 eukaryotic proteins recognized as containing a SPRY domain in the SMART database,^11^ with 46 encoded in the human genome. More than half of the known SPRY domains have a conserved N-terminal extension (PRY domain), which, together with the SPRY domain, creates the B30.2 domain.^12,13^ However, in proteins such as SPSB2 that do not contain a PRY domain, this motif is replaced by a similar structural domain with no obvious sequence homology to the PRY motif, even though such proteins are often referred to simply as SPRY domains; thus, at the structural level, PRYSPRY domains could be regarded as a subset of SPRY domains,^14^ even though current nomenclature would suggest the converse. Three-dimensional structures of several B30.2/SPRY domain-containing proteins have been published recently.^5,10,14,15–18^ Structural comparisons also reveal similarity to two structures of Neuralized homology repeat domains (also known as NEUZ domains).^14,19^

The specific functions of B30.2/SPRY domain-containing proteins encoded by the human genome are poorly characterized, but our understanding is expected to increase greatly with the identification of their respective interaction partners. All reported B30.2/SPRY domain structures have a broadly similar β-sandwich core but show differences in the surface-exposed regions, consistent with their binding to diverse sets of ligands. The GUSTAVUS complex with VASA maps the binding site to five variable loops (designated A–E) on the opposite face of the SPRY domain to the C-terminal SOCS box, while the primary binding epitope of VASA is the hexapeptide sequence DINNNN. In *Drosophila*, this interaction is required for the localization of VASA to the posterior pole of the developing oocyte, with GUSTAVUS mutations causing female sterility.^9^ SPSB1 and SPSB4 are similarly proposed to regulate germ cell physiology, showing expression in the mouse ovary in granulosa cells at all stages of follicular development.^20^ However, the VASA epitope is absent in the human orthologue and other ligands await identification. Importantly, GUSTAVUS and human SPSB1 (hSPSB1) are competent to bind the similar ELNNNL sequence of hPar-4, suggesting that the hSPSB family proteins will share a conserved recognition mode in their substrates.^10^

In this article, we investigate the potential conservation of the peptide binding mode in the murine and hSPSB family proteins. NMR spectroscopy was employed to screen for additional residues in hPar-4 that participate in SPSB binding, and the contributions of key positions were analyzed by mutagenesis and binding affinity measurements. Crystal structures were then solved for hSPSB1 in complex with hPar-4 and VASA, the hSPSB2 complex with VASA, and apo-hSPSB4, in order to understand the significant differences in their relative affinities and preferred peptide sequences.

## Results

### Interaction of hPar-4 peptides with mSPSBs via ITC

The interaction of Par-4 with SPSB family members was investigated initially using murine SPSB (mSPSB) proteins because of the availability of mSPSB2 mutants^5^ and a set of NMR assignments.^21^ Isothermal titration calorimetry (ITC) measurements showed that hPar-4_(59–77)_ bound to mSPSB2-elonginBC with low micromolar affinity (*K*_d_ = 4.3 μM) (Table 1) and to mSPSB4-elonginBC more strongly (*K*_d_ = 214 nM). It has been shown in an affinity purification assay that the interactions of hPar-4 with mSPSB1, mSPSB2, and mSPSB4 are mediated via the SPRY domain.^5^ Our data confirmed that the absence of the SOCS box and elonginBC did not affect binding affinities, with mSPSB2ΔSB and mSPSB4ΔSB showing *K*_d_ values with hPar4_(59–77)_ of 4.6 μM and 189 nM, respectively (Table 1).

mSPSB1 and mSPSB4 bound the shorter hPar-4 peptide, hPar-4_(64–77)_, tightly, with *K*_d_ values of 210 and 213 nM, respectively, whereas mSPSB2 again showed only low micromolar affinity (*K*_d_ = 3.1 μM) (Table 1). Overall, the relative affinities are consistent with the higher sequence identity of mSPSB1 with SPSB4 (75%) compared to mSPSB2 (55%). The additional five N-terminal residues in hPar-4_(59–77)_ make no significant contribution to the binding affinity for mSPSB2 or mSPSB4.

Mutation to Ala of the conserved residues Tyr120 and Val206 in the loop regions of mSPSB2 (Supplementary Fig. S1) reduced the binding affinity for hPar-4_(59–77)_ to the point where it could not be quantified by ITC. Similarly, the triple mutants [L123A,L124A,G125A] and [S126A,N127A,S128A] showed no binding to hPar-4_(59–77)_ according to ITC (Table 1). The effect of mutation of Thr198 was tested to explore the extent of the hPar-4 binding surface (Fig. S1); the [T198A] mutant still bound hPar-4_(59–77)_, exhibiting a *K*_d_ of 3.0 μM.

Mutation of Asn72 in hPar-4_(59–77)_ abolished binding to mSPSB2 and mSPSB4 (Table 1). This concurs with a previous study that demonstrated poor binding of [N72A]hPar-4_(67–81)_ to hSPSB1-elonginBC^10^ and confirms that Asn72 is one of the key residues mediating interactions with the SPSB proteins. Mutations of Asn70 and Asn71 to Ala in the shorter hPar-4 peptide hPar-4_(64–77)_ also abolished binding to mSPSB1, mSPSB2, and mSPSB4 according to ITC (Table 1). For the double mutant [E68D,L69I]hPar-4_(64–77)_ (which provides hPar-4 with a DINNN motif similar to VASA), the binding affinities for mSPSB1 and mSPSB4 increased by 5- to 6-fold, while the affinity for mSPSB2 increased by 30-fold (Table 1). Subsequently, we mutated each of the two residues of the double mutant [E68D,L69I]hPar-4_(64–77)_ to establish which residues were important for the interactions. The affinities of [E68D]hPar-4_(64–77)_ and [L69I]hPar-4_(64–77)_ to mSPSB2 increased by 15- and 2-fold, respectively, compared to wild type. In contrast, only small differences were observed in the binding affinities of [E68D]hPar-4_(64–77)_ and [L69I]hPar-4_(64–77)_ for mSPSB4 compared with the double mutant (Table 1). Mutation N-terminal to the predicted interface did not affect binding, with [A66D]hPar-4_(59–77)_ having wild-type affinities for mSPSB2 and mSPSB4.

### Structure of hPar-4_(59–77)_

hPar-4_(59–77)_ is largely unstructured in aqueous solution, consistent with predictions and experimental data for the full-length protein.^22^ This is evident from the ^1^H^–^^15^N heteronuclear single quantum coherence (HSQC) spectrum that shows limited peak dispersion in the ^1^H dimension (Fig. S2). More than half the residues of hPar-4_(59–77)_ showed minimal deviations from random-coil amide ^1^H shifts,^23^ the exceptions being the N- and C-terminal residues Thr60, Ala62, Gly75, Gly76, and Ala77 (Fig. S3a). Similarly, only the N- and C-terminal residues, as well as Asn67 and Leu73, showed chemical shift differences from random coil in the ^15^N dimension (Fig. S3b).^24^

### Identification of key interacting residues of hPar-4_(59–77)_ and mSPSB2

**Fig. 1:**
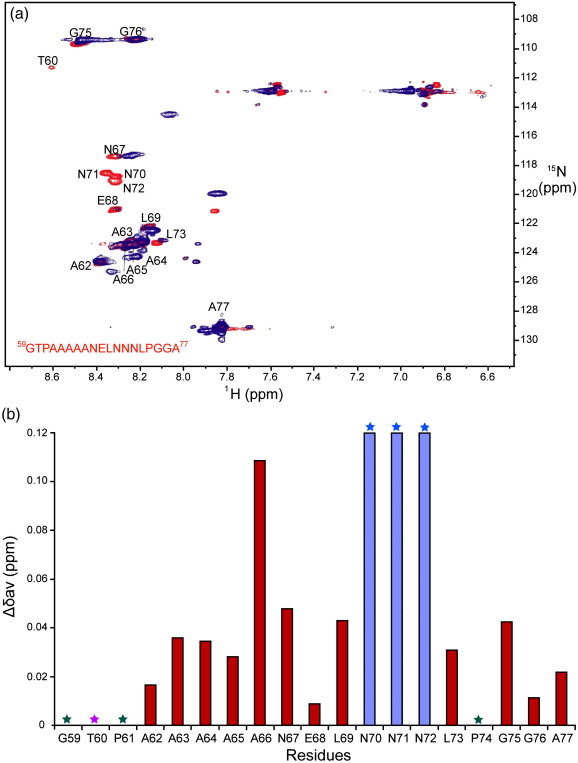
Identification of key interacting residues of hPar-4. (a) Comparison of HSQC spectra of uniformly ^15^N-labeled hPar-4_(59–77)_, free (red) and in a 1:1 complex with mSPSB2_(12–224)_ (blue). Spectra were recorded on 0.1-mM solutions in 95% H_2_O/5% ^2^H_2_O, pH 6.7, and 295 K, using a Bruker Avance500 spectrometer with a cryoprobe. (b) Weighted average chemical shift variations of ^15^N and ^1^H between free and bound forms of ^15^N-labeled hPar-4_(59–77)_. The weighted averages of the three Asn residues, Asn70–72 (blue asterisks and blue bars), are a representation only, as the peaks in the complex may have shifted more than indicated. Green asterisks correspond to residues (Gly59, Pro61, and Pro74) not observed in these spectra, while the purple asterisk denotes a very weak peak from Thr60.

NMR interaction studies were performed by saturating 104 μM ^15^N-hPar-4_(59–77)_ with 380 μM mSPSB2, resulting in 97% of ^15^N-hPar-4_(59–77)_ being bound to mSPSB2_(12–224)_. Peaks for Asn70-Asn72 of ^15^N-hPar-4_(59–77)_ could not be identified unambiguously after saturation with mSPSB2_(12–224)_ as these resonances shifted significantly from their positions in free hPar-4; peaks at ^1^H/^15^N chemical shifts of 7.84 ppm/120.0 ppm and 8.06 ppm/114.5 ppm are likely candidates for these resonances in the bound form. Peaks corresponding to Ala62, Ala66, Asn67, Leu69, Leu73, Gly75–76, and Ala77 were perturbed (Fig. 1a), indicating that they are either involved directly in the interaction or affected indirectly by binding. The weighted averages of perturbations in ^15^N and ^1^H chemical shifts (Δδav) upon binding are plotted in Fig. 1b.

**Fig. 2:**
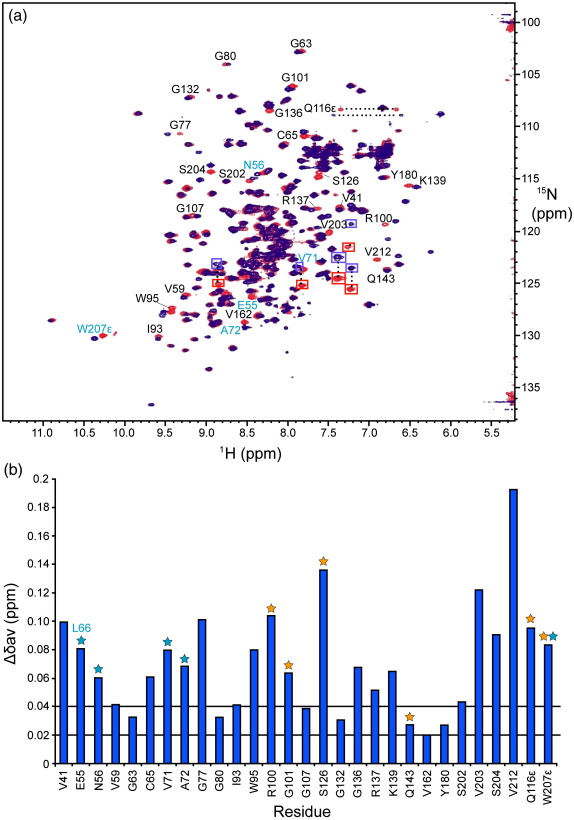
Identification of key interacting residues of mSPSB2. (a) Comparison of HSQC spectra of 0.1 mM uniformly N-labeled mSPSB2_(12–224)_, free (red) and in a 1:1 complex with hPar-4_(59–77)_ (blue). Spectra were recorded in 95% H_2_O/5% ^2^H_2_O, pH 6.7, 295 K, at 500 MHz. The red peaks are labeled with sequence-specific assignments for free mSPSB2_(12–224)_ using the one-letter code and sequence positions (black). Conserved residues of GUSTAVUS and three SPSB proteins that are important for GUSTAVUS/VASA^10^ and mSPSB2/hPar-4^5^ interactions are represented in cyan. Aliased resonances arising from Arg side chains of mSPSB2 are shown in red (free) and blue (complex) square boxes as these were due to different spectra widths used in the ^15^N dimension. (b) Weighted average chemical shift variations of ^15^N and ^1^H between free and bound forms of ^15^N-labeled mSPSB2_(12–224)_. Cyan asterisks represent those residues that are conserved in GUSTAVUS, mSPSB1, mSPSB2, and mSPSB4 and have been shown to be involved in GUSTAVUS binding to VASA,^10^ except for mSPSB2 E55, where L66 is found in GUSTAVUS, mSPSB1, and mSPSB4. Orange asterisks indicate those residues that are known to be involved in mSPSB2/hPar-4 interaction according to a previous study.^5^ Horizontal lines show cutoffs for weighted average chemical shift differences of 0.02  and 0.04 ppm. The Trp207 peak is from the indole NH; the backbone amide resonance for this residue is close to the water resonance^5,21^ and difficult to follow.

Unlabeled hPar-4_(59–77)_ was then added to 96 μM ^15^N-mSPSB2_(12–224)_ to a final concentration of 227 μM, resulting in 99% of ^15^N-mSPSB2_(12–224)_ being bound to hPar-4_(59–77)_. HSQC spectra of free ^15^N-mSPSB2_(12–224)_ (red) and ^15^N-mSPSB2_(12–224)_ with hPar-4_(59–77)_ bound (blue), including the side chains of Gln116 and Trp207, are superimposed in Fig. 2a. Aliased resonances arising from Arg side chains of mSPSB2 are shown in red (free) and blue (complex) square boxes. There were approximately 22 residues with weighted average chemical shift variations > 0.04 ppm and a further 7 with > 0.02 ppm (Fig. 2b). Peaks from conserved residues of GUSTAVUS and three SPSB proteins that were recognized to be important for GUSTAVUS/VASA^10^ and mSPSB2/hPar-4 interactions^5^ are labeled in cyan in Fig. 2a. The five residues involved in GUSTAVUS binding to VASA are denoted by cyan asterisks in Fig. 2b and the six involved in the mSPSB2/hPar-4 interaction are denoted by orange asterisks. Key interacting residues are mapped onto the mSPSB2 structure in Fig. S4.

### Crystal structures of hSPSB1, SPSB2, and SPSB4

**Fig. 3:**
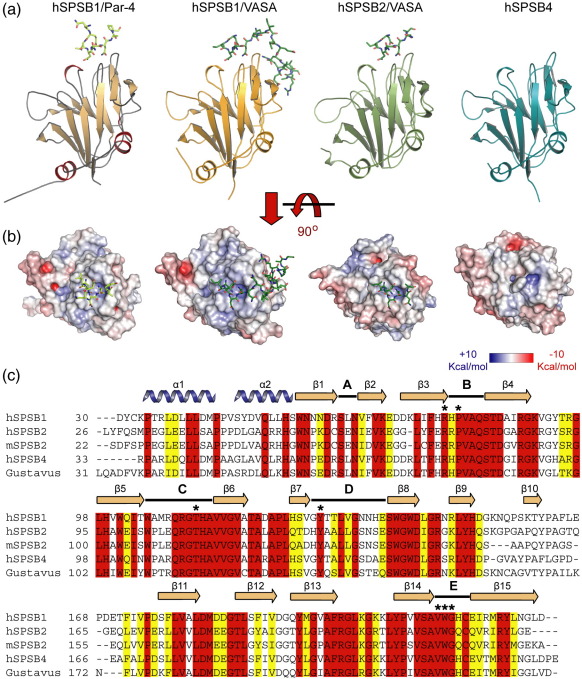
Crystal structures of hSPSB1, SPSB2, and SPSB4. (a) Overview of the structures of hSPSB1 in complex with hPar-4 and VASA, as well as the hSPSB2 complex with VASA and apo-hSPSB4. Peptide ligands are shown as sticks. (b) Molecular surface representation of hSPSB structures. The electrostatic surface potential is conserved across the hPar-4 binding site, but more varied beyond this epitope. (c) Sequence alignment of SPRY domain structures in the SPSB family. Secondary-structure elements are shown for hSPSB1, including binding loops A–E. Key hPar-4 contact residues are indicated by asterisks. Red and yellow shading indicate positions with sequence identity and sequence similarity, respectively. An enhanced 3D visualization file displaying all structures is available for download (file S6).

To characterize the binding interface of SPSB proteins in more detail, we performed extensive crystallographic studies using the SPRY domains of hSPSB1–4 together with the cognate substrate peptide from hPar-4_(67–81)_ and the VASA_(184–203)_ peptide from *Drosophila*. ITC studies on these human proteins not only confirmed the weaker binding to hPar-4 of hSPSB2 relative to hSPSB1 and hSPSB4 but also revealed the far tighter binding of all three proteins to the VASA-derived peptide (Table 2). For example, the hSPSB2 affinity for VASA is 10-fold greater (*K*_d_ = 147 nM) than that for hPar-4. High-resolution structures were solved for hSPSB1 in complexes with hPar-4 and VASA, as well as the hSPSB2/VASA complex and the apo-hSPSB4 protein (see Table 3 for data collection and refinement statistics; see Fig. S5 for electron density maps). All three hSPSB proteins share the same conserved SPRY domain fold with two short N-terminal helices packed against a bent β-sandwich, comprising two 7-stranded β-sheets (Fig. 3). The N- and C-terminal parts of the domain pack together and, by similarity with GUSTAVUS [Protein Data Bank (PDB) ID: 2FNJ], are expected to support the C-terminal SOCS box. The opposite side of the β-sandwich supports five extended loops (designated A–E) that form the previously identified interaction site for VASA (PDB ID: 2IHS).^10^

### Comparison of VASA binding to hSPSB1 and GUSTAVUS

The structure of hSPSB1 in complex with VASA was refined to 2.05 Å resolution and contained two copies of the protein complex in the asymmetric unit. Overall, hSPSB1 residues 30–233 and VASA residues 184–201 were well defined. The two hSPSB1 molecules superimpose with a main-chain r.m.s.d. of 0.38 Å, with deviations observed in only the N-terminus and the long loop connecting β10–β11 (both distant from the peptide binding site). Superimposition of GUSTAVUS (2IHS) and hSPSB1 shows similar sites of divergence, with a main-chain r.m.s.d. of 1.08 Å, reducing to 0.45 Å upon exclusion of these same regions. The VASA peptide is bound to GUSTAVUS and hSPSB1 in the same extended manner, with the central loop B of the protein dissecting the peptide into N- and C-terminal interaction sites. Between these sites, the central VASA residues Ile191, Val192, and Glu193 make little or no contact with the protein. The C-terminal peptide residues were poorly defined in the density, with only Asp194 and Glu196 forming significant interactions with hSPSB1 Asn64 and Leu63, respectively. This explains previous ITC studies^10^ showing that the VASA binding affinity is retained by a six-residue peptide (184–189, DINNNN) derived from the N-terminal interaction site. The VASA contact residues in hSPSB1 and GUSTAVUS are conserved across both sites, highlighting the importance of this interface, and both complexes formed with similar affinity (*K*_d_  ∼ 40 nM). Consequently, we limit further description of the VASA interface to its comparison with hPar-4.

### Conserved binding mode of hPar-4

**Fig. 4:**
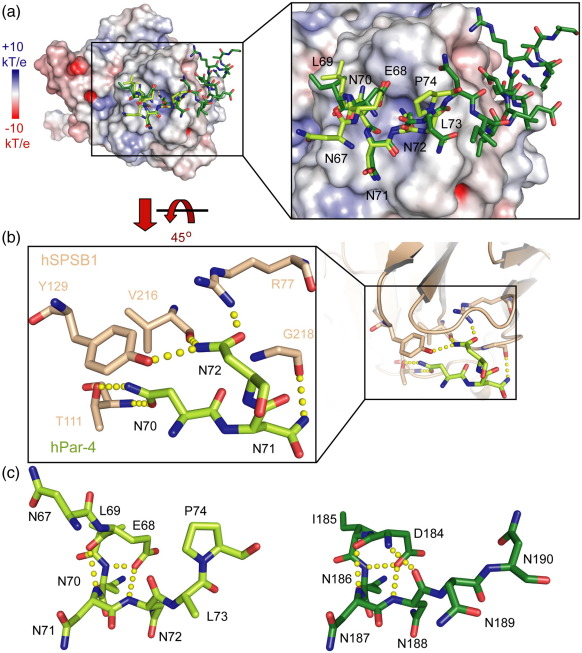
Comparison of the VASA and hPar-4 binding modes to hSPSB1. (a) Overlay of the hSPSB1 structures in complex with VASA (dark green) and hPar-4 (light green). The surface of hSPSB1 is colored by electrostatic surface potential. The peptide NNN motifs bind a shallow pocket centered on hSPSB1 Arg77. The peptide backbone deviates at hPar-4 Glu68 where the shorter Asp184 side chain of VASA enables an intramolecular main-chain hydrogen bond with VASA Asn188. (b) Stick representation showing the side-chain hydrogen bonds formed by the central hPar-4 NNN motif. Similar bonds are formed in the VASA complex. (c) Intramolecular hydrogen bonding in hPar-4 and VASA. The VASA conformation is stabilized by an additional main-chain hydrogen bond between Asp184 and Asn188.

The structure of hSPSB1 bound to hPar-4_(67–81)_ was refined to 1.79 Å resolution, enabling direct comparison of the hPar-4 and VASA binding modes in hSPSB1 (Fig. 4a). Residues Asn67-Pro74 from hPar-4 were well defined, corresponding to the minimal binding motif determined by NMR chemical shift changes. Further electron density was not present to build the C-terminal residues (75–81, GGAPAAP), suggesting that, as for VASA, the N-terminal site is sufficient for high-affinity interaction. The hPar-4 and VASA peptides adopt similar conformations, with tight superimposition of the conserved NNN motifs, which form a total of six conserved hydrogen bonds, including two between hPar-4 Asn70 and hSPSB1 Thr111 (side and main chain), one between Asn71 and the Gly218 carbonyl, and three from Asn72 to Arg77, Tyr129, and the Val216 carbonyl (Fig. 4b). One further conserved main chain–main chain hydrogen bond is formed between Asn70 of hPar-4 and the Gly218 amide of hSPSB1. Notably, this position is deeply buried by the close packing on either side of Tyr129 and Trp217 from hSPSB1 loops D and E, respectively.

The main-chain conformation of hPar-4 deviates from that of VASA on both sides of the NNN motif. Towards the N-terminus, the Glu68 carboxyl moiety of hPar-4 overlays well with VASA Asp184 to hydrogen bond with hSPSB1 Tyr129 (equivalent to SPSB2 Tyr120) and the three main-chain amides of the NNN motif. This position is stabilized in VASA by a 2. 6-Å intramolecular hydrogen bond formed between the amide of Asp184 and the main-chain carbonyl of Asn188. However, the longer Glu68 side chain of hPar-4 reorients the backbone to replace this bond with the somewhat unfavorable 3.3-Å juxtaposition of the Glu68 β-carbon with the hPar-4 Asn72 carbonyl (Fig. 4c). At the N-terminus, the hPar-4 residue Asn67 makes no contact with hSPSB1. To the C-terminus of the NNN motif, the introduction of a proline residue (Pro74) also induces a main-chain shift; in VASA, the preceding residue, Asn189, makes both main-chain amide and carbonyl hydrogen bonds to the hSPSB1 backbone, but in hPar-4, only the amide bond can form. Neither Leu73 nor Pro74 in hPar-4 makes a significant contribution to binding.

### Comparison of hSPSB1 and hSPSB2

**Fig. 5:**
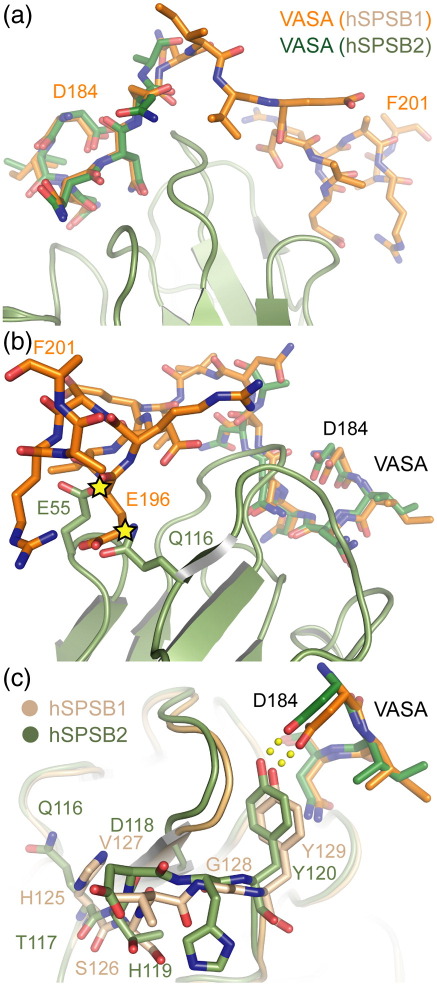
Comparison of the hSPSB1 and hSPSB2 binding modes. Overlay of the hSPSB1 (orange) and hSPSB2 (green) structures in complex with VASA. (a) The N-terminal region of VASA in both structures superimposes well, but the C-terminal region was disordered in the hSPSB2 structure. The hSPSB2 SPRY domain is shown in green ribbon. (b) Overlay of the two structures reveals a likely steric clash between the VASA C-terminal region (orange) and the side chains of hSPSB2 Glu55 and Gln116 (green). (c) hSPSB1 and hSPSB2 show structural changes in β7/loop D where hSPSB1 residues 125-HSVG-128 are replaced by hSPSB2 116-QTDH-119. At a neighboring position, only a small change is observed in the position of the hSPSB2 Tyr120 hydrogen bond with VASA Glu196, with little change in the overall bond length or geometry.

The hPar-4 binding residues in hSPSB1 are completely conserved in hSPSB2 and yet the binding of hSPSB2 to hPar-4 is weak (*K*_d_ = 1.5 μM). Perhaps as a result, we were able to crystallize hSPSB2 in complex with the higher-affinity VASA peptide but not the hPar-4 peptide. Nonetheless, this provides a valuable structure for comparison with the hSPSB1–peptide structures (Fig. 5). The weaker binding of hSPSB2 has been noted previously and was predicted to result from amino acid changes in β7 and loop D, where 116-QTDH-119 replace hSPSB1 residues 125-HSVG-128.^10^ In particular, the substitution of hSPSB1 Gly128 was expected to distort the neighboring tyrosine, which in hSPSB1 (Tyr129) forms critical hydrogen bonds with Glu68 and Asn72 in hPar-4, as well as hydrophobic contacts. Indeed, mutation of these four positions to HSVG improved the hSPSB2 affinity for hPar-4, albeit by only fourfold.^10^

The most significant difference in the hSPSB2/VASA structure is the complete lack of electron density for the C-terminal residues of the peptide: only residues Asp184-Asn190 are defined, compared to Asp184-Phe201 in the equivalent hSPSB1 structure. Overlay of the two structures reveals a likely clash between VASA Glu196 and hSPSB2 residues Glu55 and Gln116 (Fig. 5b). The N-terminal region of the peptide is otherwise bound through identical interactions, with conservation of all interface positions. The amino acid substitutions from Gln116-His119 do induce a subtle conformational change in β7 and loop D. For example, the φ angle of hSPSB1 Gly128 changes from + 72° to − 164° for the equivalent hSPSB2 residue His119. However, changes to the neighboring hSPSB2 Tyr120 are minimal, with the lengths and angles of its hydrogen bonds to VASA Asp184 and Asn188 being essentially unchanged (Fig. 5c). It appears that further conformational changes are accommodated by local readjustment across the entire peptide binding site. Indeed, hSPSB2–peptide interactions are associated with a higher entropic penalty than those of hSPSB1 and hSPSB4 (Table 2). Interestingly, the hSPSB2-bound VASA peptide overlays closely with the hPar-4 backbone at its visible C-terminus and similarly can make only one main-chain hydrogen bond from Asn189 as the carbonyl and neighboring Asn190 are shifted away from the protein surface. As expected, structural conservation with hSPSB1 is greatest at the peptide NNN motif and the loop D tryptophan that supports it (Trp217 in hSPSB1 and Trp207 in hSPSB2). This site appears unchanged by the neighboring substitution of hSPSB1 His219 with hSPSB2 Gln209.

### Structural changes upon ligand binding

**Fig. 6:**
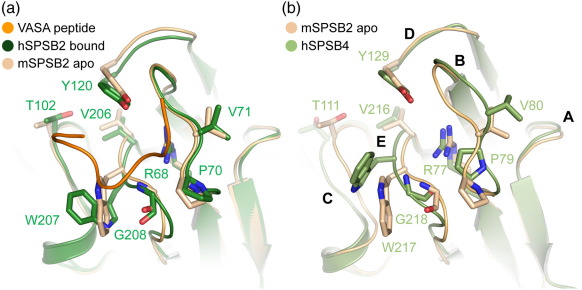
Structural changes upon ligand binding. (a) Overlay of the hSPSB2/VASA complex and apo-mSPSB2 structure.^14^ Side-chain rearrangements between the ligand-free and ligand-bound state are largely limited to hSPSB2 Trp207. The hSPSB2-bound VASA peptide is shown in ribbon representation for reference and colored orange. (b) Overlay of the ligand-free structures of hSPSB4 and mSPSB2 reveals more substantial structural changes in loops B, C, and E. The side chain of hSPSB4 Trp217 also shows a substantial rotamer change from mSPSB2 Trp207. Note that this residue has an unusual backbone amide chemical shift in apo-mSPSB2.^5^

Comparison of the apo-mSPSB2 structure (PDB ID: 3EK9^14^) and the hSPSB2/VASA complex reveals only modest loop rearrangements upon binding (Fig. 6a). Moieties forming hydrogen bonds with the peptide and those involved in hydrophobic interactions reorient slightly to optimize those interactions. An exception is the side-chain indole of hSPSB2 Trp207, which is significantly rotated upon peptide binding to pack against the side chain of VASA Asn187. The identical conformation of this Trp in all hSPSB1, hSPSB2, and GUSTAVUS peptide complexes suggests that a rearrangement of this side chain is essential for binding, in particular to alleviate a steric clash that would otherwise arise from the apo conformation.

The apo-structures of mSPSB2 and hSPSB4 show some additional differences in loop conformation (Fig. 6b). The most significant change affects loop B, where hSPSB4 Pro79 and Val80 are displaced relative to the corresponding residues in mSPSB2. However, the orientation of the adjacent hSPSB4 Arg77 residue, which would bind the third Asn (hPar-4 Asn72), is essentially unchanged because of its largely buried position in the β3 strand. Loops C (e.g., hSPSB4 Thr111) and E (e.g., hSPSB4 Trp217) are also somewhat displaced relative to mSPSB2, although their overall conformations are similar. Again, a significant rearrangement of the Trp side chain would be required to accommodate the peptide ligand.

The lack of significant structural change upon peptide binding is intriguing in light of previously published NMR relaxation data and coarse-grained dynamics simulations of mSPSB2,^25^ which found evidence for flexibility in some loops of the protein on the picosecond-to-nanosecond timescale (although not on submillisecond-to-millisecond timescales). Perhaps these higher-frequency motions were already dampened significantly in the crystal structure of apo-mSPSB2.

## Discussion

The human proteins SPSB1–4 are expected to form a subfamily of cullin5 E3 ligases comprising a SPRY domain and C-terminal SOCS box. To date, substrate recognition sites for the SPRY domain are identified only for hPar-4 (ELNNNL) and the *Drosophila* orthologue GUSTAVUS binding to a peptide from VASA (DINNNN). The sequences of these peptides suggest that further SPSB substrates may be identified from the consensus motif (E/D)(L/I)NNN. To further investigate this consensus, we have determined the first structures of the human proteins SPSB1, SPSB2, and SPSB4 as well as their binding modes and affinities for both hPar-4 and VASA.

The structures show remarkable conservation of the SPRY domain fold and absolute sequence conservation for positions in contact with the (E/D)(L/I)NNN motif. For such a limited core motif, the low nanomolar binding affinities of SPSB1 and SPSPB4 are extraordinary, reflecting the highly satisfied hydrogen bonding and complementary packing interactions. Despite the sequence conservation, the majority of hydrogen bonds from the SPRY domain originate from the main chain. In fact, the contribution to the binding efficiency from side chain–side chain hydrogen bonds is largely restricted to Arg77 (loop B) and Tyr129 (loop D), although the packing interactions are critically dependent on Trp217 and Gly218 (hSPSB1 numbering). Notably, all four residues are absent in hSPSB3, explaining its lack of binding to hPar-4. The structural variation in the loops of other SPRY domain families also suggests that their specificities will differ.

Given the restricted recognition motif and apparent conservation of structure and interactions, two key observations remain in question. Firstly, why is Asp preferred over Glu at the start of the motif, and secondly, why is the binding affinity of SPSB2 far inferior to those of SPSB1 and SPSB4? The preference for Asp at the first position is evident both from the greater affinity of VASA compared to hPar-4 and from the similar affinity increase provided by the direct substitution of this residue into the lower affinity hPar-4 peptide. Comparable hSPSB1 structures in complex with VASA and hPar-4 reveal identical positions of the Asp and Glu carboxyl, but the shorter Asp side chain affords an additional intramolecular main-chain hydrogen bond. This further stabilizes the bound conformation of the peptide and provides a rationale for the more favorable Asp interaction.

The effect of the Glu-to-Asp change is significantly greater for SPSB2 than for SPSB4. This highlights the hSPSB2 Tyr120 interaction with the Glu/Asp side chain as a potential cause for its weak binding. The substitution of hSPSB1 Gly128 to His119 in hSPSB2 flips the geometry of the backbone carboxyl for the preceding residue, Asp118, producing a subtle change in the hSPSB2 loop position. This is reflected in an apparent shift of the Tyr120 position in a structural overlay of the VASA complexes with hSPSB1 and hSPSB2 (Fig. 5c). Indeed, substitutions within β7/loop D have been shown previously to increase hSPSB2 affinity for hPar-4 by fourfold.^10^ In the structure of the hSPSB2/VASA complex, these changes also prevent interaction of the VASA C-terminal segment, and a main-chain hydrogen bond is lost from SPSB2 Ala72 to the fourth Asn repeat residue Asn189.

Substitution of these hSPSB2 β7/loop D residues with those of hSPSB1 does not provide a complete rescue of hPar-4 affinity.^10^ Structural overlay of the hSPSB1 and hSPSB2 complexes guided solely by the VASA DINNN motif shows identical positioning of both the DINNN peptide residues and their hydrogen bond partners in the SPRY domain. Therefore, assigning affinity changes to individual positions (such as hSPSB2 His119) in these structures may be too simplistic, as conformational changes are dissipated across the structure, reflecting the lower sequence conservation of SPSB2 relative to SPSB1 and SPSB4 and their potentially different functions.^20,26^

The structures and affinity data presented here provide the first structural description of the hSPSB family and show the potential variation in their substrate binding modes. The findings also provide an extensive data set to challenge computational algorithms that describe protein–protein interfaces. Finally, the high affinities suggest that the (E/D)(L/I)NNN consensus provides a good basis to experimentally screen for additional SPSB family substrates, and thus achieve a greater understanding of their physiological function(s).

## Materials and Methods

### Expression and purification

Full-length mSPSB1 (SWISS-PROT accession number Q9D5L7) was cloned into a pGEX-4T vector (GE Healthcare UK Ltd., Buckinghamshire, UK) that contained human elonginB (SWISS-PROT accession number Q15370). Similarly, mSPSB2 and mSPSB4 (SWISS-PROT accession numbers O88838 and Q8R5B6, respectively) were individually cloned into a pGEX-4T vector that contained murine elonginB (SWISS-PROT accession number P62869). An N-terminally truncated murine elonginC_(17–112)_ starting at the Met17 (SWISS-PROT accession number P83940) was cloned into pBB75 vector for co-expression with mSPSB and elonginB. Full-length SPSB proteins and elonginB were co-expressed with elonginC in BL21(DE3) cells (Novagen, San Diego, CA) at 30 °C for 4 h with 0.2 mM IPTG at OD_600_ (optical density at 600 nm) = 0.6–0.8. The glutathione *S*-transferase (GST)–SPSB–elonginBC complex was purified using glutathione-Sepharose 4B (GE Healthcare, UK) and cleaved from the GST-tag with one unit of thrombin (Roche Applied Science, Germany) per 10 mg of fusion protein at 4 °C for 18 h on a rotating mixer. Flow-throughs of the cleaved reactions were collected and purified further using gel-filtration chromatography, Superdex 200 10/300 GL (GE Healthcare Bio-Sciences AB, Uppsala, Sweden). The fractions of SPSB–elonginBC complex from gel-filtration chromatography were concentrated using a 3000 molecular weight cutoff centrifugal filter device, Amicon Ultra-4 (Millipore, Billerica, MA).

Shorter mSPSB constructs were also used for mSPSB1 and mSPSB4 lacking the C-terminal SOCS box region (mSPSB1ΔSB and mSPSB4ΔSB); they were cloned into pGEX-2T (GE Healthcare, UK). The shorter mSPSB2 construct excluded the first 11 residues at the N-terminus and the C-terminus SOCS box [mSPSB2_(12–224__)_]. mSPSB2_(12–224)_ and mutants used in this study have been described previously.^21^ Each of the mSPSBΔSB constructs was expressed in Origami B(DE3) pLysS cells (Novagen, Madison, WI) as a GST fusion protein at 20 °C for 24 h with 0.5 mM IPTG added at OD_600_ = 0.6–0.8. The mSPSBΔSB proteins were isolated according to the method used for purifying the mSPSB–elonginBC complex.

Human SPSB constructs lacking the SOCS box region [hSPSB1_(24–233)_, SWISS-PROT accession number Q96BD6; hSPSB2_(26–219)_, Q99619 and hSPSB4_(28–233)_, Q96A44] were cloned into pNIC28-Bsa4 by ligation-independent cloning. Constructs were transformed into BL21(DE3)-R3-pRARE2 and expressed overnight at 18 °C with 0.5 mM IPTG added at OD_600_ = 0.5–0.8. hSPSB proteins were purified using Ni-Sepharose (GE Healthcare, UK), eluted by imidazole and subsequently cleaved from the N-terminal hexahistidine tag by addition of tobacco etch virus protease (although hSPSB2 was resistant to cleavage). Further purification was achieved by size-exclusion chromatography and finally anion-exchange chromatography in the case of hSPSB1.

The cloning, expression, and purification of hPar-4_(59–77)_ and mutants are described in the Supplementary Data.

### Synthetic hPar-4 and mutants

The shorter wild-type peptide hPar-4_(64–77)_ and the mutant peptides [N70A]hPar-4_(64–77)_, [N71A]hPar-4_(64–77)_, and [E68D,L69I]hPar-4_(64–77)_ were synthesized by GenScript Corporation (Piscataway, NJ). The [A66D]hPar-4_(59–77)_ peptide was synthesized by GL Biochem (Shanghai, China). For crystallization and ITC of hSPSB proteins, the wild-type peptides hPar-4_(67–81)_ and VASA_(184–203)_ were synthesized by ThermoElectron GmbH (Ulm, Germany).

### NMR spectroscopy

All NMR experiments were performed on a Bruker Avance500 spectrometer (Bruker BioSpin GmbH, Rheinstetten, Germany) using a TXI cryoprobe at 22 °C. All NMR samples were prepared in 95% H_2_O/5% ^2^H_2_O containing 10 mM sodium phosphate, 50 mM sodium chloride, 2 mM ethylenediaminetetraacetic acid, and 0.02% (w/v) sodium azide at pH 6.7, to a final concentration of approximately 0.1 mM, unless otherwise indicated. Three-dimensional ^15^N-edited nuclear Overhauser enhancement spectroscopy (mixing time 250 ms), ^15^N-edited total correlated spectroscopy (spin lock time, 75 ms), and ^15^N HSQC spectra were recorded on uniformly labeled ^15^N-hPar-4_(59–77)_ and ^15^N-mSPSB2_(12–224)_. The ^1^H chemical shifts were referenced indirectly to TMS at 0 ppm via the H_2_O signal, while the ^15^N chemical shifts were referenced indirectly using the ^15^N/^1^H γ-ratios.^27^ Spectra were processed using TOPSPIN version 1.3 (Bruker) and analyzed with XEASY version 1.3.^28^

### Isothermal titration calorimetry

ITC measurements on mSPSB proteins were performed at 22 °C using a MicroCalorimetry System (MicroCal, Northhampton, MA). mSPSB1, mSPSB2, and mSPSB4 were prepared by dialyzing against 20 mM Tris, 150 mM sodium chloride, and 2.5 mM calcium chloride at pH 8.0. Measurements on hSPSB proteins were performed at 20 °C using a VP-ITC titration calorimeter (MicroCal). hSPSB1 protein was dialyzed against 50 mM Hepes, pH 7.4, 150 mM NaCl, and 1 mM DTT. hSPSB2 and hSPSB4 proteins were dialyzed against 50 mM Hepes, pH 7.4, 150 mM NaCl, and 0.5 mM TCEP. Peptides for each experiment were dissolved in the corresponding dialysis buffers. ITC measurements were routinely carried out by titrating 100 μM peptide solution into 10 μM protein. Dilution enthalpies were determined separately by titrating peptide into buffer and used to correct the enthalpies of the binding of protein and peptide. Data were analyzed using a single binding site model implemented in the Origin software package provided with the instrument.

### Crystallization, data collection, and structure solution

hSPSB1 was buffered in 20 mM Hepes, pH 7.5, 100 mM NaCl, 10 mM l-arginine, 10 mM l-glutamate, and 10 mM DTT. hSPSB2 was buffered in 20 mM Hepes, pH 7.4, 150 mM NaCl, and 10 mM DTT. hSPSB4 was buffered in 50 mM Hepes, pH 7.5, 150 mM NaCl, and 5 mM DTT. Peptides were added to a final concentration of 2 mM. Crystallization was carried out using the sitting drop vapor diffusion method at 4 °C. The complex of hSPSB1/hPar-4 (I) was grown by mixing 75 nl of concentrated protein (4 mg/ml) with 75 nl of a well solution containing 0.2 M Li_2_SO_4_, 0.1 M 2-[bis(2-hydroxyethyl)amino]-2-(hydroxymethyl)propane-1,3-diol, pH 5.5, and 20% polyethylene glycol (PEG) 3350. The complex of hSPSB1/VASA (II) was grown by mixing 666 nl of the concentrated protein (4 mg/ml) with 333 nl of a well solution containing 0.2 M NaCl, 0.1 M 2-[bis(2-hydroxyethyl)amino]-2-(hydroxymethyl)propane-1,3-diol, pH 5.5, and 25% PEG 3350. The complex of hSPSB2/VASA (III) was grown by mixing 666 nl of the concentrated protein (9 mg/ml) with 333 nl of a well solution containing 0.22 M Na/KPO_4_, 11% ethylene glycol, and 22% PEG 3350. Apo-hSPSB4 (IV) was grown by mixing 100 nl of protein with 50 nl of a solution containing 14.4% PEG 10 K, 0.16 M Ca(ac)_2_, 20% glycerol, and 0.08 M cacodylate, pH 6.5. In all cases, crystals grew to diffracting quality within a few days and were cryo-protected using the well solution supplemented with 25% additional ethylene glycol and were flash frozen in liquid nitrogen.

Data were collected at a Rigaku FRE Superbright equipped with an RAXIS IV detector at 1.5 Å (I), or at the Swiss Light Source beamline PX10 in the case of (II) (at 1.00001 Å) and (IV) (at 0.9537 Å), or at the Diamond Synchrotron beamline I03 in the case of (III) (at 0.9756 Å). Indexing and integration was carried out using MOSFLM^29^ and scaling was performed with SCALA^30^ for (I), (II), and (III) or XDS^31,32^ and SHELX^33^ for (IV). Initial phases for (I) and (IV) were calculated by molecular replacement with PHASER^34^ using 2FNJ as a starting model. Initial phases for (II) were calculated likewise using an ensemble comprising 2FNJ, 2V24, and 2IHS starting models. The models were completed manually in Coot^35^ and were refined with REFMAC5.^34^ The final model of (I) was used to obtain phases for (III) followed by manual build in Coot^35^ and refinement with REFMAC5.^34^ In all cases, thermal motions were analyzed using TLSMD^36^ and hydrogen atoms were included in late refinement cycles. Data collection and refinement statistics can be found in Table 3.

### Accession numbers

The models and structure factors have been deposited with PDB accession codes: 2JK9 (I), 3F2O (II), 3EMW (III), and 2V24 (IV).

### Note added in proof

A paper in press in The Journal of Cell Biology (Kuang, Z. *et al.*, The SPRY domain–containing SOCS box protein SPSB2 targets iNOS for proteasomal degradation. *J. Cell Biol.* In press. doi:10.1083/jcb.200912087) identifies SPSB2 as a novel negative regulator of inducible nitric oxide synthase that recruits an E3 ubiquitin ligase complex to polyubiquitinate iNOS, resulting in its proteasomal degradation. SPSB2 binds via a DINNN motif in the N-terminus of iNOS.

## Figures and Tables

**Table 1:** ITC analysis of hPar-4 peptide interactions with mSPSB proteins

Ligands	Proteins	*K*_d_^a^
hPar-4_(59–77)_ GTPAAAAANELNNNLPGGA	mSPSB2_(12–224)_	4.6 ± 0.1 μM
	mSPSB4ΔSB	189 ± 1 nM
	mSPSB2 + elonginBC	4.3 ± 0.6 μM
	mSPSB4 + elonginBC	214 ± 36 nM
	mSPSB2_(12–224)_[Y120A]	nm^b^
	mSPSB2_(12–224)_[123–5]	nm
	mSPSB2_(12–224)_[126–8]	nm
	mSPSB2_(12–224)_[T198A]	3.0 ± 0.2 μM
	mSPSB2_(12–224)_[V206A]	nm
[N72A]hPar-4_(59–77)_ GTPAAAAANELNNALPGGA	mSPSB2_(12–224)_	nm
	mSPSB4 + elonginBC	nm
hPar-4_(64–77)_ AAANELNNNLPGGA	mSPSB2_(12–224)_	3.2 ± 0.002 μM
	mSPSB4ΔSB	213 ± 7 nM
	mSPSB1ΔSB	210 ± 19 nM
[N70A]hPar-4_(64–77)_ AAANELANNLPGGA	mSPSB2_(12–224)_	nm
	mSPSB4ΔSB	nm
	mSPSB1ΔSB	nm
[N71A]hPar-4_(64–77)_ AAANELNANLPGGA	mSPSB2_(12–224)_	nm
	mSPSB4ΔSB	nm
	mSPSB1ΔSB	nm
[E68D,L69I]hPar-4_(64–77)_ AAANDINNNLPGGA	mSPSB2_(12–224)_	106 ± 30 nM
	mSPSB4ΔSB	33 ± 23 nM
	mSPSB1ΔSB	40 ± 5 nM
[E68D]hPar-4_(64–77)_ AAANDLNNNLPGGA	mSPSB2_(12–224)_	208 ± 13 nM
	mSPSB4ΔSB	47 ± 22 nM
[L69I]hPar-4_(64–77)_ AAANEINNNLPGGA	mSPSB2_(12–224)_	1.6 ± 0.4 μM
	mSPSB4ΔSB	55 ± 8 nM
[A66D]hPar-4_(59–77)_ GTPAAAADNELNNNLPGGA	mSPSB2_(12–224)_	4.0 ± 0.9 μM
	mSPSB4ΔSB	255 ± 5 nM

a *K*_d_ value deviations are shown as the standard error of the mean.

b nm, not measurable because * K*_d_ > 50 μM.

**Table 2:** ITC analysis of peptide interactions with hSPSB proteins

Protein:Peptide	*K*_d_^a^ (nM)	*N* (stoichiometric ratio)	Δ*H*_binding_ (kcal/mol)	*T*Δ*S*_binding_ (kcal/mol)
hSPSB1:hPar-4^b^	150 ± 12	0.94	− 13.2 ± 0.1	− 4.1 ± 0.1
hSPSB1:VASA^c^	40 ± 5	0.96	− 20.5 ± 0.2	− 10.6 ± 0.2
hSPSB2:hPar-4	1520 ± 50	1.18	− 16.2 ± 0.1	− 8.4 ± 0.1
hSPSB2:VASA	147 ± 7	0.96	− 23.9 ± 0.1	− 14.7 ± 0.1
hSPSB4:hPar-4	65 ± 4	1.19	− 17.0 ± 0.1	− 7.4 ± 0.1
hSPSB4:VASA	4.0 ± 0.7	0.97	− 25.2 ± 0.1	− 13.9 ± 0.1

a Deviations represent the errors of the curve fit to the data obtained using the single binding site model used within the Origin software provided with the instrument.

b hPar-4_(67__–81)_ has sequence NELNNNLPGGAPAAP.

c VASA_(184__–203)_ has sequence DINNNNNIVEDVERKREFYI.

**Table 3:** Data collection and refinement statistics

Protein	hSPSB1/hPar-4 (I)	hSPSB1/VASA (II)	hSPSB2/VASA (III)	hSPSB4 (IV)
*Data collection*				
PDB ID	2JK9	3F2O	3EMW	2V24
Space group	*P*2_1_	*P*2_1_2_1_2_1_	*P*2_1_2_1_2_1_	*P*3_2_21
Cell dimensions				
*a*, *b*, *c* (Å)	37.52, 82.96, 38.54	75.67, 80.65, 86.86	34.47, 61.96, 118.58	104.22, 104.22, 40.78
α, β, γ (°)	90.0, 104.42, 90.0	90.0, 90.0, 90.0	90.0, 90.0, 90.0	90.0, 90.0, 120.0
Resolution (Å)	1.79 (1.89–1.79)	2.05 (2.16–2.05)	1.80 (1.90–1.80)	2.20 (2.30–2.20)
Unique observations	20,682 (2853)	34,014 (4899)	24,366 (3494)	13,208 (1635)
Completeness (%)	96.2 (91.4)	99.9 (100.0)	99.9 (100.0)	99.9 (100.0)
Redundancy	3.5 (3.3)	3.6 (3.5)	3.5 (3.5)	10.9 (10.9)
*R*_merge_	0.066 (0.536)	0.092 (0.649)	0.078 (0.618)	0.070 (0.560)
*I*/σ*I*	12.7 (2.0)	10.0 (2.0)	15.0 (3.1)	18.7 (3.4)

*Refinement*				
Resolution (Å)	1.79	2.05	1.80	2.20
*R*_work_/*R*_free_ (%)	19.2/23.9	18.0/23.2	17.8/21.6	20.9/26.0
Number of atoms (protein/other/water)	1671/0/121	3490/0/287	1583/16/138	1533/1/78
*B*-factors (Å^2^) (protein/other/water)	24.75/—/24.62	32.79/—/31.26	28.98/41.44/37.63	38.23/21.06/34.57

Values in parentheses correspond to the highest-resolution shell.

## Appendix A

Revised Supplementary Data

File S6. Enhanced 3D visualization of the hSPSB crystal structures. This is the enhanced 3D visualization of the hSPSB crystal structures in which text (extracted from the main manuscript) is integrated with 3D representations and animated transitions (triggered via the links in the text). The 3D graphics display window is fully interactive, allowing the reader to quickly explore any scene by rotating, translating and zooming. Interactive 3D visualization of the hSPSB crystal structures. Users should download the .zip file and extract the .icb file; free ICM-Browser software is required to access the .icb file. This interactive view has been created using the iSee concept and ICM programs.^38,39^
