# Supplementary material for: Structural Basis for Par-4 Recognition by the SPRY Domain- and SOCS Box-Containing Proteins SPSB1, SPSB2, and SPSB4
Source: J Mol Biol. 2010 Aug 20;401(3-2):389–402. doi: 10.1016/j.jmb.2010.06.017 (PMC2923778; doi:10.1016/j.jmb.2010.06.017)
Supplement: Supplementary file 1 — Revised Supplementary Data [file mmc1.pdf]

Supplementary data

Structural basis for Par-4 recognition by the SPRY domain and SOCS box containing proteins SPSB1, SPSB2 and SPSB4

Panagis Filippakopoulos[a†], Andrew Low[b†], Timothy D. Sharpe[a], Jonas Uppenberg[a], Shenggen Yao[b], Zhihe Kuang[b], Pavel Savitsky[a], Rowena S. Lewis[b], Sandra E. Nicholson[b], Raymond S. Norton[b]*, Alex N. Bullock[a]*

[a]*Structural Genomics Consortium, University of Oxford, Old Road Campus, Roosevelt Drive, Oxford OX3 7DQ, UK*
[b]*The Walter and Eliza Hall Institute of Medical Research, 1G Royal Parade, Parkville, Victoria 3052, Australia*

**Corresponding authors:*
Raymond S Norton; email: rnorton@wehi.edu.au; Address: The Walter and Eliza Hall Institute of Medical Research, Structural Biology, 1G Royal Parade, Parkville 3052, Australia; Tel: +61 3 9345 2306; Fax: +61 3 9345 2686.
Alex N. Bullock; email: alex.bullock@sgc.ox.ac.uk; Address: Structural Genomics Consortium, University of Oxford, Old Road Campus, Roosevelt Drive, Oxford OX3 7DQ, UK; Tel: +44 (0)1865 617754; Fax: +44 (0)1865 617575.

[†]PF and AL contributed equally to this work.

Supplementary Methods

Expression and Purification

The gene corresponding to residues 59-77 of full-length human Par-4 was amplified by PCR amplification and cloned into the pET32a vector (Novagen, Madison, WI) via EcoRI/HindIII sites. The plasmid was transformed into BL21-CodonPlus™ (DE3)-RP cells (Stratagene, La Jolla, CA). A similar technique was also used to generate the [N72A]hPar-$4_{(59\text{-}77)}$ mutant gene. Unlabelled hPar-$4_{(59\text{-}77)}$ and ^{15}N-labelled hPar-$4_{(59\text{-}77)}$ were expressed as thioredoxin-tagged fusion proteins in BL21-CodonPlus™ (DE3)-RP cells (Strategene) in 2 l of Superbroth with 2 g/l glucose or 2 l of M9 minimal medium containing 1 g/l $^{15}NH_4Cl$ and 4 g/l glucose, respectively [37]. The fusion protein was expressed mainly as soluble protein at 30 °C for 4 h and purified initially using chelating Sepharose (GE Healthcare Bio-Sciences AB, Uppsala, Sweden), where approximately 0.2 g of fusion protein per litre of culture was isolated. One thousand units of TEV (Invitrogen, Carlsbad, CA) was used to cleave 10 mg of fusion protein at 4 °C for 24 h on a rotating mixer, resulting in the separation of thioredoxin-S-tag and hPar-$4_{(59\text{-}77)}$. The cleavage mixture was loaded onto a RP-HPLC column (10 µm column, 10 mm x 250 mm C8 Polymeric-Reverse-Phase, 300 Å pore size) (GraceVydac, Hesperia, CA) and a gradient of 0 to 50 % acetonitrile, ChromAR HPLC grade (Mallinckrodt Baker Inc, Philipsburg, NJ) and 0.085 % (v/v) trifluoroacetic acid, HPLC/Spectro grade (Pierce, Rockford, IL) over 30 min at a flow rate of 3 ml/min was used to separate hPar-$4_{(59\text{-}77)}$ from the cleavage mixture, with hPar-$4_{(59\text{-}77)}$ eluting at 27.5 % acetonitrile. [N72A]hPar-$4_{(59\text{-}77)}$ was expressed and purified in a similar manner to its wild-type counterpart. After cleavage and RP-HPLC, yields of hPar-$4_{(59\text{-}77)}$ and [N72A]hPar-$4_{(59\text{-}77)}$ were approximately 20 mg/l of culture. The corresponding yield for ^{15}N-labelled hPar-$4_{(59\text{-}77)}$ was 15 mg.

Supplementary references

37. Low, A., Chandrashekaran, I. R., Adda, C. G., Yao, S., Sabo, J. K., Zhang, X., Soetopo, A., Anders, R. F. & Norton, R. S. (2007). Merozoite surface protein 2 (MSP2) of Plasmodium falciparum: Expression, structure, dynamics and fibril formation of the conserved N-terminal domain. *Biopolymers* **87**, 12-22.

Figure S1. **Mapping of mutations on mSPSB2 that affect binding to hPar-4.** The crystal structures of hPar-4$_{(67-74)}$ (PDB code 2JK9) and mSPSB2$_{(12-224)}$ (PDB code 3EK9) were each subjected to positional restrained molecular dynamics simulations on the solvent and unrestrained molecular dynamics simulations on the whole system via GROMACS. (**A**) The resulting model shows mSPSB2 as a white ribbon and hPar-4$_{(67-74)}$ as a yellow ribbon. Residues whose mutation resulted in decreased affinity of mSPSB2 for hPar-4 (Table 1 and ref 5) are shown in blue and labelled. The Thr198Ala mutation (shown in magenta) did not affect the mSPSB2/hPar-4 interaction. Mutation of residues shown in orange (Leu123, Leu124, Gly125) affected binding to both hPar-4 (Table 1 and c-Met [5]); in the case of hPar-4 at least, this effect may be mediated via conformational changes at the hPar-4 binding site caused by these mutations. (**B**) A 45° rotation along the y-axis of (A).

We thank Dr Brian J. Smith for guidance in the molecular dynamics refinement

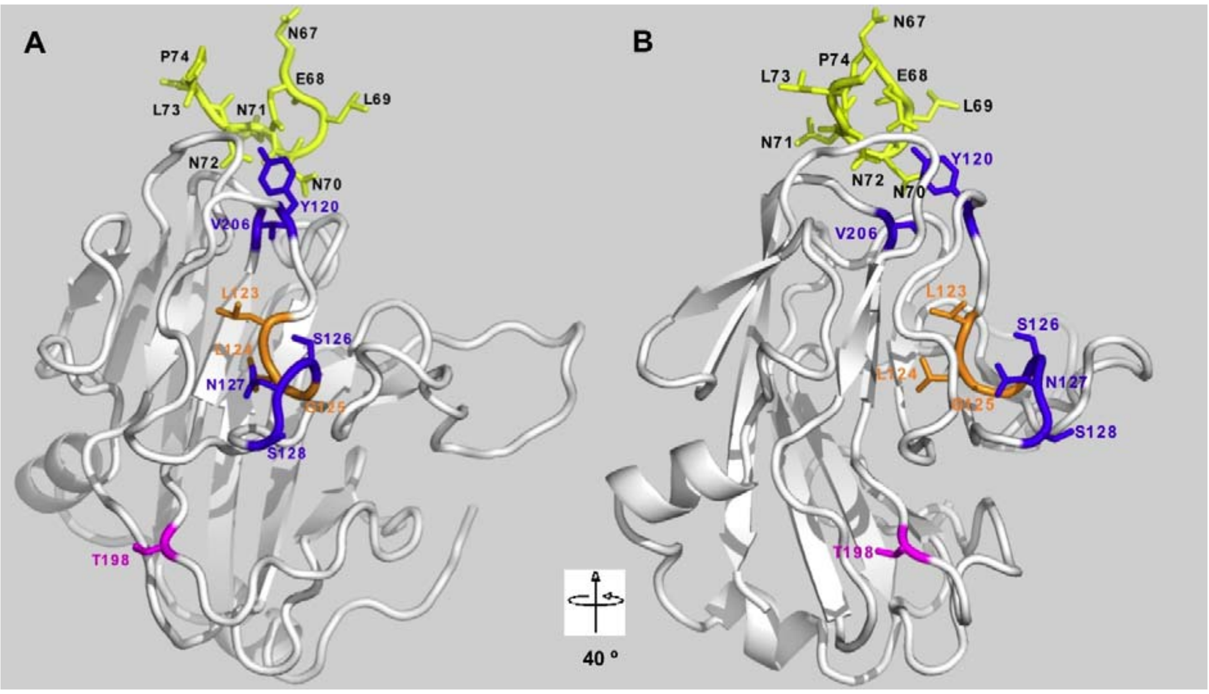

Figure S2. **HSQC spectrum of hPar-4 in solution**. ^{1}H-^{15}N HSQC spectrum of uniformly ^{15}N-labelled hPar-4$_{(59\text{-}77)}$ at 0.1 mM in 95% H_2O/5% $^{2}H_2O$, pH 6.7, 295 K, recorded using a Bruker Avance500 spectrometer with a cryoprobe. Red peaks are labelled with the sequence-specific assignments for free hPar-4$_{(59\text{-}77)}$ using single-letter code and the sequence positions (black). Minor forms of the hPar-4 peptide are labelled in green boxes. The hPar-4 sequence is shown in red at bottom left. Resonances from only 16 of the 17 residues (excluding the two Pro) are observed in this spectrum as the resonance from the first residue is not visible under these conditions. Resonances from minor forms of a few residues were observed when the contour level threshold was decreased to reveal the weak Thr60 peak; *cis-trans* isomerism of the peptide bonds preceding the two Pro residues is most likely to be responsible for the minor species.

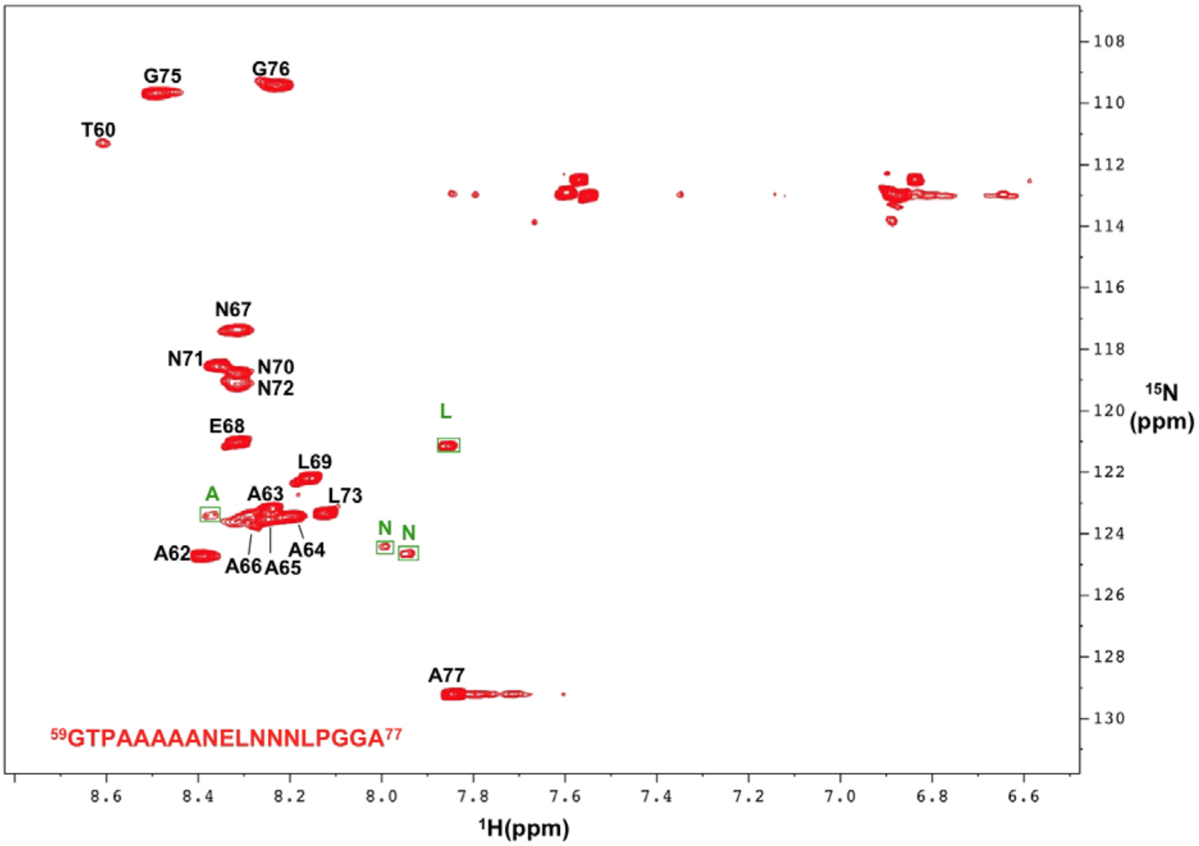

Figure S3. **hPar-4 chemical shift deviations from random coil.** (**A**) ^{1}H chemical shift deviations from random coil (Δδ) for hPar-$4_{(59\text{-}77)}$. The residues are represented in single-letter amino acid according to the residue number of full-length hPar-4. (**B**) ^{15}N chemical shift deviations from random coil (Δδ) for hPar-$4_{(59\text{-}77)}$. ^{1}H and ^{15}N chemical shifts in hPar-$4_{(59\text{-}77)}$ were compared to the random coil values of Merutka et al. [23] and Wishart et al. [24,27], respectively.

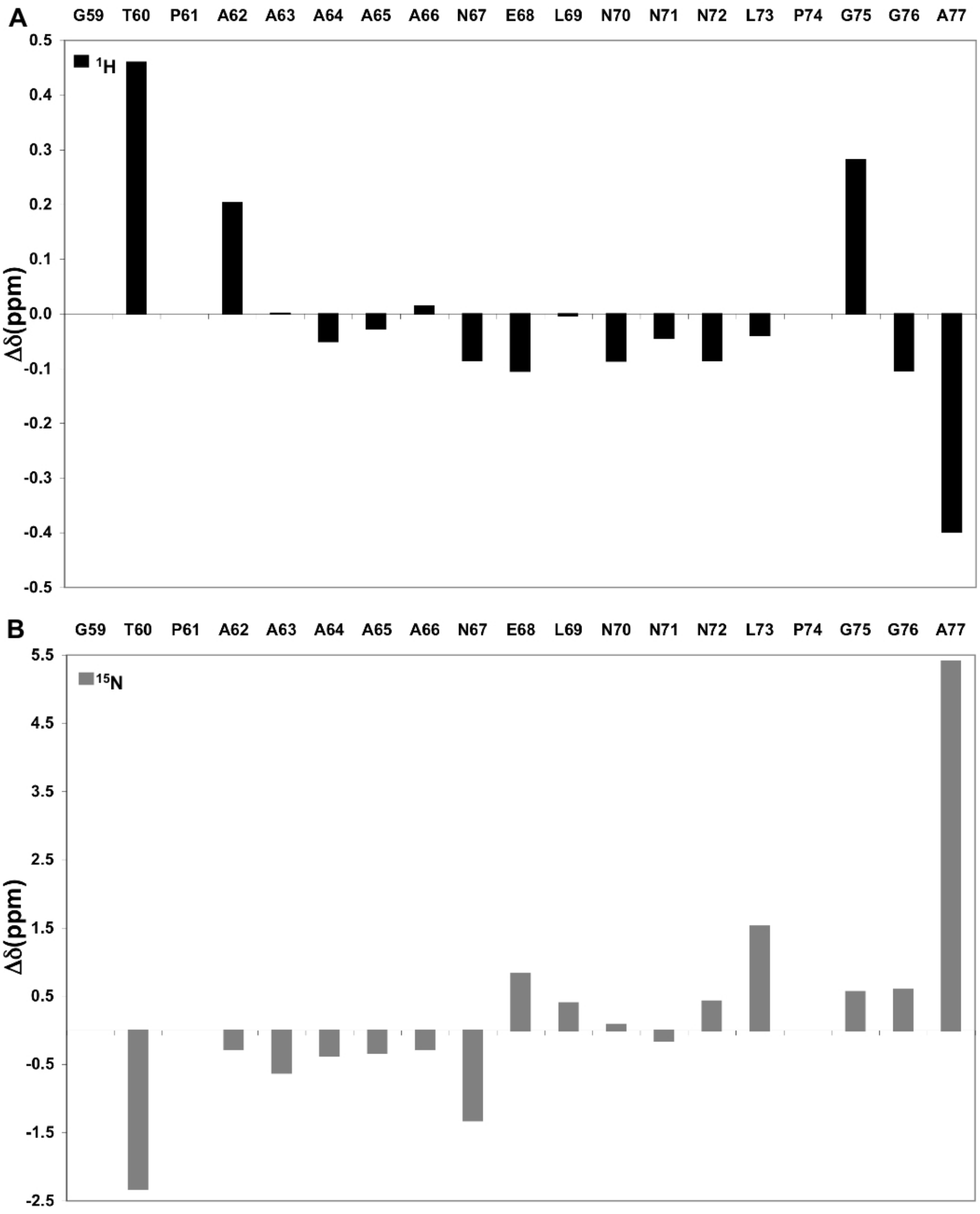

Figure S4. **Mapping of key interacting residues of hPar-4 on mSPSB2.** (A) mSPSB2/hPar-4 model from Figure S1 displaying mSPSB2 in a white surface representation and hPar-4 as yellow ribbon. The 22 mSPSB2 residues with >0.04 ppm chemical shift change upon binding of hPar-$4_{(59\text{-}77)}$ were mapped onto a shallow pocket on the mSPSB2 surface and coloured in cyan, purple and red. Cyan indicates residues conserved in mSPSB2 and GUSTAVUS that were affected by the binding of hPar-$4_{(59\text{-}77)}$ (Figure 2) and VASA [10], respectively. Purple residues were affected by hPar-4 binding according to mutational analysis (Table 1 [5]), and red residues were only affected by the binding of hPar-$4_{(59\text{-}77)}$ in the current NMR titration study. (B) A 45º rotation along the y-axis of (A). NMR titration of hPar-$4_{(59\text{-}77)}$ induced chemical shift changes in some mSPSB2 residues (Arg100 and Arg137) that were not affected by the shorter hPar-$4_{(67\text{-}74)}$ peptide. These residues border a pocket formed between loops C and D (shown by an arrow) that may provide flexibility for these residues to shift as a consequence of conformational changes in mSPSB2 associated with hPar-$4_{(59\text{-}77)}$ binding.

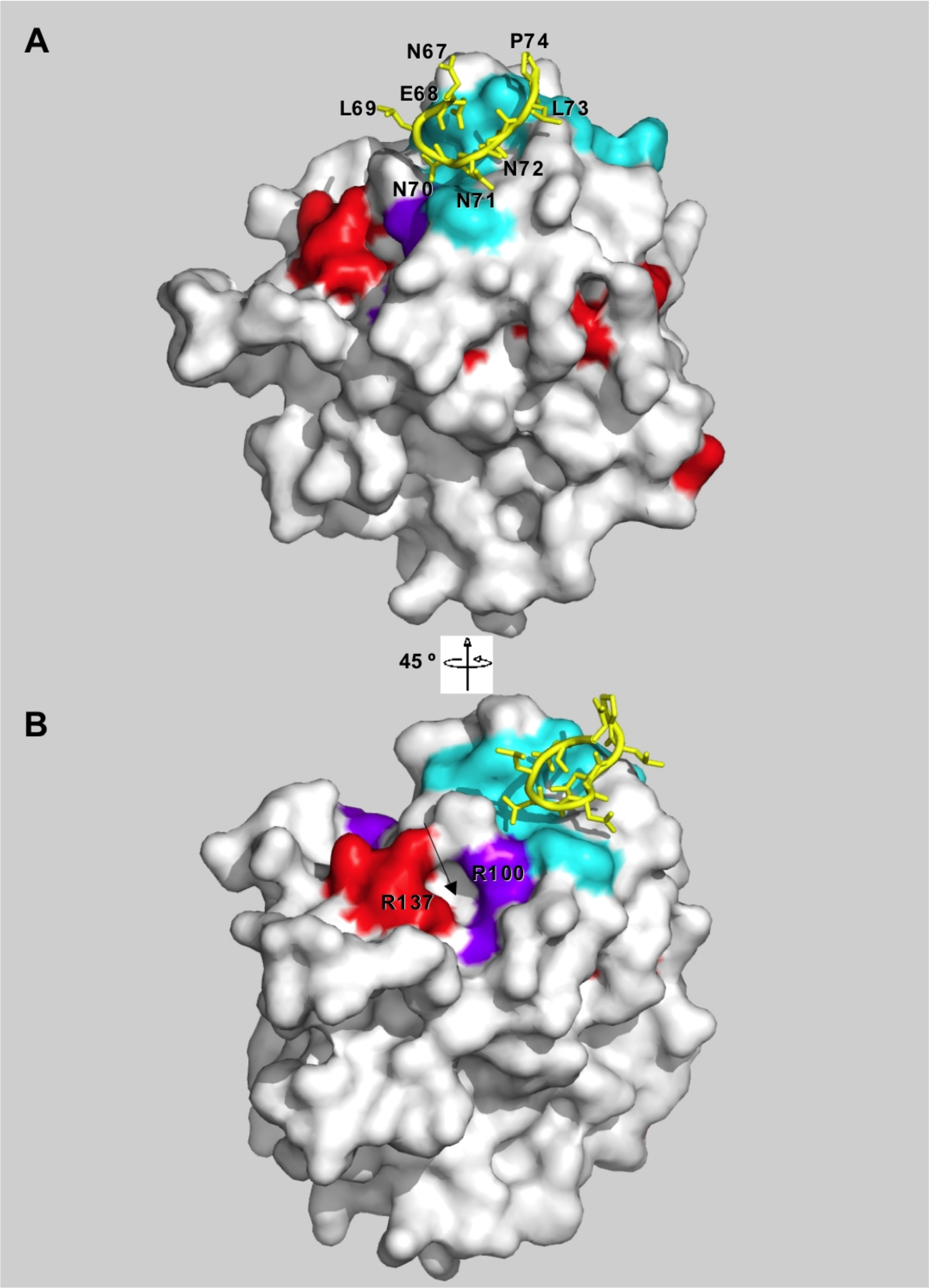
A
N67
P74
E68
L69
L73
N72
N70
N71
45 º
B
R100
R137

Figure S5. Electron density maps (2Fo-2Fc) contoured at 2σ for the bound peptides in the hSPSB1 and hSPSB2 structures.

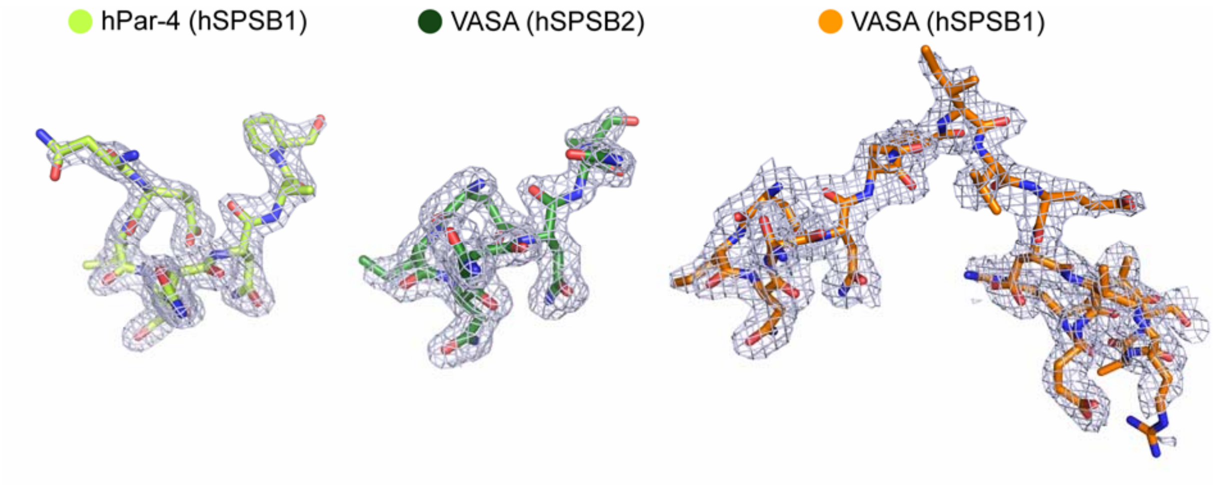

Supplementary visualization

File S6. Enhanced 3D visualization of the hSPSB crystal structures. This is the enhanced 3D visualization of the hSPSB crystal structures in which text (extracted from the main manuscript) is integrated with 3D representations and animated transitions (triggered via the links in the text). The 3D graphics display window is fully interactive, allowing the reader to quickly explore any scene by rotating, translating and zooming. Interactive 3D visualization of the hSPSB crystal structures. Users should download the .zip file and extract the .icb file; free ICM-Browser software is required to access the .icb file. This interactive view has been created using the iSee concept and ICM programs.[38,39]

Supplementary references

38. Lee, W. H., Atienza-Herrero J., Abagyan, R., Mariden, B. D. (2009). SGC-structural biology and human health: a new approach to publishing structural biology results. *PLoS One* **4**(10), e7675. doi:10.1371/journal.pone.0007675.

39. Raush, E., Totrov, M., Marsden, B. D., Abagyan, R. (2009). A new method for publishing three-dimensional content. *PLoS One* **4**(10), e7394. doi:10.1371/journal.pone.0007394.
